# Agonism of FFA4/GPR120 activates tyrosine hydroxylase and confers neuroprotection from 6-OHDA-induced cytotoxicity in PC12 cells and in a rat 6-OHDA model of Parkinson’s disease

**DOI:** 10.1016/j.bcp.2026.117685

**Published:** 2026-01-06

**Authors:** Farnoosh Moghaddam, Monika Binwal, Andrea J. Green, Kalyn M. Rambacher, Bo Jarrett Wood, Kevin S. Murnane, Nader H. Moniri

**Affiliations:** a Department of Pharmaceutical Sciences, College of Pharmacy, Mercer University Health Sciences Center, Mercer University, Atlanta, GA 30341, USA; b Department of Pharmacology, Toxicology & Neuroscience, Louisiana State University Health Sciences Center at Shreveport, Shreveport, LA 71103, USA; c Louisiana Addiction Research Center, Louisiana State University Health Sciences Center at Shreveport, Shreveport, LA 71103, USA; d Department of Psychiatry and Behavioral Medicine, Louisiana State University Health Sciences Center at Shreveport, Shreveport, LA 71103, USA; e Department of Biomedical Sciences, School of Medicine, Mercer University Health Sciences Center, Mercer University, Macon, GA 31207, USA

**Keywords:** FFA4, GPR120, Free-fatty acids, Tyrosine hydroxylase, Dopamine, Parkinson’s disease

## Abstract

Free-fatty acid receptor 4 (FFA4/GPR120), a G-protein-coupled receptor activated by medium to long-chain free-fatty acids (FFA) including ω-3 polyunsaturated fatty acids, regulates many metabolic and anti-inflammatory processes, and has also been linked to diverse neurophysiological functions. Here, we investigated the expression and functional role of FFA4 in dopaminergic systems using PC12 cells, rat striatal tissue, and an *in vivo* 6-hydroxydopamine (6-OHDA) model of Parkinson’s disease (PD). FFA4 mRNA and protein were expressed in rat caudate-putamen and colocalized with tyrosine hydroxylase (TH), the rate-limiting enzyme in dopamine biosynthesis. Agonism of FFA4 by the endogenous agonist docosahexaenoic acid (DHA) or the selective synthetic agonist TUG-891 significantly increased phosphorylation of TH-Ser^40^ in PC12 cells and rat striatal minces, without elevating cAMP, suggesting non-canonical signaling of FFA4 to TH. In PC12 cells, TUG-891 attenuated 6-OHDA-induced cytotoxicity in a concentration-dependent manner, concomitant with reductions in reactive oxygen species (ROS) generation and NF-κB transcriptional activity, effects that were reversed by the FFA4 antagonist AH-7614. In an *in vivo* rat 6-OHDA model of PD, TUG-891 administration significantly mitigated apomorphine-induced rotational asymmetry and motor deficits in 6-OHDA-lesioned rats, while preserving striatal TH immunoreactivity. These findings identify FFA4 as a novel modulator of dopaminergic neuron integrity that may operate through antioxidant and anti-inflammatory signals to preserve cell function, warranting further exploration of FFA4 in dopaminergic degeneration and PD.

## 1. Introduction

Parkinson’s Disease (PD) is characterized by motor symptoms including bradykinesia, tremor, motor rigidity, and postural instability, which arise due to degeneration of dopamine (DA) producing neurons of the substantia nigra pars compacta (SNc). Recent research has also underscored the significance of non-motor symptoms, including cognitive and executive dysfunction and psychiatric manifestations, as well as peripheral alterations, such as disruption of gastrointestinal function in PD [1,2]. Although the etiology of this neurodegeneration remains unclear, oxidative stress and neuroinflammation in the micro-environment of the substantia nigra play important roles in facilitating DA neuron cell death [3,4]. For nearly six decades, levodopa or DA receptor agonists have been the standards for pharmacological treatment of PD, yet these only alleviate motor symptoms without affecting the underlying neuroinflammation, cell death or disease progression.

Tyrosine hydroxylase (TH) is the rate-limiting enzyme in the biosynthesis of DA, and its activity is regulated in a complex manner primarily via N-terminal phosphorylation, as well as by DA-mediated feedback inhibition and cysteine-oxidation [5,6]. Notably, DA synthesis is tightly regulated by TH phosphorylation, and catalytic activity of the enzyme is primarily controlled by the phosphorylation state of Ser^40^, which is essential towards the phosphoregulation of other residues in the N-terminus, including Ser^31^ [7–11]. While homeostatic DA concentrations in the SNc are required for coordinated movement, DA is also inherently highly oxidative and its metabolism and quinone-containing metabolites facilitate oxidative stress, including formation of reactive oxygen species (ROS) such as H_2_O_2_, that can drive mitochondrial dysfunction, synthesis and aggregation of α-synuclein, and dysregulation of TH [6,12], all of which likely contribute to the pathology of PD.

Dietary free-fatty acids (FFA), including n-3 (ω3) fats such as α-linolenic acid (ALA), eicosapentaenoic acid (EPA), and docosahexaenoic acid (DHA), have a long record of linkage to brain health and physiological function and are major lipid constituents of neuronal membranes [13]. They have also been shown to have neuroprotective roles in neuronal cell culture and animal models of neurodegeneration, including PD [14]. We have previously demonstrated that DHA induces TH activity and DA synthesis, is neuroprotective of DA neuron loss, and preserves motor function in *in vivo* 6-hydroxydopamine (6-OHDA) models of PD in rodents [15]. Traditional models propose that ω3 fatty acids exert their effects primarily through non-selective mechanisms, such as intracellular metabolism or stabilization of neuronal membranes, where they serve as major structural components. However, recent research has revealed that a family of lipid-sensitive cell surface G protein-coupled receptors (GPCR), termed free-fatty acid receptors (FFAR), mediate distinct, receptor-dependent actions of ω3 fatty acids that exhibit FFA preference, cell type-specificity, and are amenable to modulation by medicinal chemistry [16–19]. This discovery positions FFAR as druggable targets, enabling the development of nanomolar-potent, receptor-selective synthetic ligands designed to maximize therapeutic efficacy while minimizing off-target effects [13]. In the present study, we identify one such FFAR, free-fatty acid receptor-4 (FFA4, previously termed GPR120), which is expressed broadly within the brain and across multiple peripheral tissues [16,20–24] and has recently been shown to be expressed and functionally active in dopaminergic neurons [25], as a novel regulator of TH activation and dopaminergic neuroprotection. Using DA-producing PC12 cells, rat striatal minces, and 6-OHDA lesioned rats, here, we investigate the effects of FFA4 agonism on TH activity, cellular resilience to oxidative and inflammatory stress, and *in vivo* neuroprotection against DA degeneration relevant to PD pathology.

## 2. Materials and methods

### 2.1. Reagents and chemicals

TUG-891 and AH-7614 were purchased from Tocris (Minneapolis, MN), DHA was purchased from Nu-Chek Prep (Elysian, MN). 3-isobutyl-1-methylxanthine (IBMX) and forskolin were from Sigma-Aldrich (St. Louis MO). L-012 was from Wako Chemicals USA (Richmond, VA). All other chemicals used were obtained at the highest available purity from Thermo Fisher Scientific (Waltham, MA) or Sigma-Aldrich.

### 2.2. Cell culture and striatal minces

PC12 cells (ATCC, Manassas, VA) were cultured in 100 mm tissue culture plates containing Kaighn’s Modified F12 media with 15 % horse serum and 5 % fetal bovine serum in a humidified atmosphere of air:CO_2_ (95:5%) at 37 °C. Cells were subcultured by scraping. The NF-κB-reporter expressing stable cell line were generated as we have described previously [26]. Rat striatal minces were prepared as we have described previously from male Sprague-Dawley rats (ca. 125–150 g; Charles River, Boston, MA) [15]. Animal use was approved and overseen by the Mercer University Institutional Animal Care and Use Committee (Approval protocols A1507013, A1808012, A2501004).

### 2.3. Stereotaxic 6-OHDA lesioning

The role of FFA4 in regulating TH and promoting neuroprotection in nigrostriatal neurons was investigated using a 6-OHDA rat model of PD. Male Sprague-Dawley rats (200–225 g) were housed under standard laboratory conditions (12-h light/dark cycle, ad libitum food and water, environmental enrichment) and randomly assigned to four groups: Vehicle control (N = 5), Sham lesioned with saline (N = 6), 6-OHDA lesioned (N = 8), and 6-OHDA lesioned with daily treatment with 3 mg/kg (IP) TUG-891 (N = 8). This concentration of TUG-891 was based on its receptor potency (~100 nM) and *in vivo* rodent studies that typically utilize 1–30 mg/kg of the agonist when administered IP [27–29]. Stereotaxic surgeries were performed under isoflurane anesthesia (2.5 % induction; 1.5–2.0 % maintenance, 0.8 L/min O_2_). The left medial forebrain bundle (MFB) was targeted using coordinates from bregma (AP − 3.5 mm, ML − 2.0 mm, DV − 9.0 mm) and 6-OHDA (6 μg/μL in 0.9 % saline with 0.2 % ascorbic acid) was infused at 1 μL/min for 4 min, followed by a 5-min dwell time before needle withdrawal. Postoperatively, rats received carprofen (5 mg/kg, SQ) and cefazolin (1 mg/kg, SQ) for 2 days and were monitored daily for health, weight, and wound recovery. Beginning one day after surgery, the treatment group received daily intraperitoneal TUG-891 (3 mg/kg in 8 % Tween-80, 8 % PEG, 4 % DMSO, 80 % PBS) for 14 days, while other groups received the vehicle on the same schedule.

### 2.4. Motor coordination

Behavioral testing was performed in an unblinded manner starting 14 days after 6-OHDA injection to evaluate motor function. Briefly, rotational behavior was assessed by apomorphine (3 mg/kg, IP), with contralateral (rightward) rotations counted over one-minute periods. Balance was assessed on a 1-meter round, 3-centimeter diameter balance beam and the number of paw slips and the time to traverse the beam were recorded across three consecutive trials. At the study endpoint, animals were euthanized by CO_2_ asphyxiation followed by cervical dislocation, and brain tissues were collected for subsequent analyses.

### 2.5. TH immunofluorescence

Following perfusion with ice-cold PBS (pH 7.4) and 4 % paraformaldehyde (PFA), brain tissue was fixed in 4 % PFA, cryoprotected in 30 % sucrose, embedded in optimal cutting temperature compound, and frozen on dry ice. Coronal striatal sections (30 μm; +1.3 mm to 0.0 mm from bregma) were sectioned on a cryostat (−20 °C), mounted on Superfrost Plus slides, permeabilized in 0.3 % Triton X-100/PBS, and blocked for 1 h in 5 % normal donkey serum. Samples were incubated overnight at 4 °C with anti-tyrosine hydroxylase (TH; 1:1000, Cell Signaling Technology, Danvers, MA #2792) followed by Alexa Fluor 594 donkey anti-rat IgG (1:500, A21209, Life Technologies, Waltham, MA) for 1 h at room temperature. Nuclei were counterstained with DAPI, and slides were mounted using antifade medium, sealed with coverslips and imaged using an Echo Revolve fluorescent microscope.

### 2.6. RNA extraction, PCR and quantitative real-time PCR

Total cellular RNA was extracted from cells using the RNeasy Mini Kit (Qiagen, Gaithersburg, MD), according to the manufacturer’s directions. Extracted RNA was reverse-transcribed to cDNA using high-capacity cDNA Reverse Transcription (Applied Biosystems, Foster City, CA) and random primers. cDNA templates were amplified for 30 cycles by polymerase chain reaction (PCR) with primers as indicated. Quantitative real-time PCR was performed using EvaGreen PCR master mix (Biotium, Fremont, CA) and performed on a QuantStudio5 thermal cycler. Glyceraldehyde 3-phosphate dehydrogenase (GAPDH) was used as a control housekeeping gene with the below primers: FFA4 (N-terminal region): 5′-AGCAC-CAGCCGGTGGTCGCCC-3′ (FWD) and 5′-ATGTCCCCTGAATGCGCGCGCGGG-3′ (REV). GAPDH: 5′-ACCACAGTCCATGCCATCAC-3′ (FWD) and 5′-TCCACCACCCTGTTGCTG-TA-3′ (REV). For data analysis, the ΔΔCt method was used.

### 2.7. ROS generation

The kinetic generation of ROS was monitored by the ROS-sensitive luminol-enhanced chemiluminescent probe L-012, exactly as we have reported previously [26]. Briefly, 5.0 × 10^4^ cells were seeded in white 96 well culture plates and allowed to adhere 24 h and serum starved for 16 h. Cells were treated as indicated, rinsed three times with HBSS, then 100 μL of dye free DMEM containing 25 mM HEPES was added to each well and incubated at 37 °C for 10 min with 100 μL of L-012 (100 μM). To induce ROS generation, 6-OHDA (100 μM) in PBS was added to wells via injector and luminescence was measured on a Berthold Mithras LB940 for 15 min.

### 2.8. NF-κB reporter assay

NF-κB activity was assessed using stably transfected cells expressing the pNFκB-MetLuc2-Reporter, exactly as we have reported previously [26]. Briefly, cells were seeded in 100 mm plates and transiently transfected with 5 μg pNFκB-MetLuc2-Reporter vector, according to manufacturer’s protocol (Takara Bio USA, San Jose, CA). After 24 h, cells were plated in 24 well-plates and treated with TUG-891 (3 μM) or DHA (100 μM) in the presence or absence of 6-OHDA (1 μM) for 6 h. This concentration was used given the toxicity of higher concentrations of 6-OHDA over this period, which is required to facilitate transcription and translation of the NF-κB luciferase-tagged reporter. When AH-7614 was used, it was added 30 min prior to either agonist at a concentration of 50 μM. After 6 h, the cell culture medium was collected and analyzed utilizing for secreted luciferase in a Berthold Mithras LB940. Data are expressed as a percentage of the 6-OHDA response.

### 2.9. Immunoblotting

Immunoblotting was performed as we have described previously [15,26,30,31]. Briefly, rat striata or PC12 cells (1 × 10^6^/well) were homogenized and lysates were prepared in RIPA buffer (50 mM Tris–HCl, 150 mM NaCl, 5 mM EDTA, 1 % nonidet P-40, 0.5 % sodium deoxycholate, 0.1 % SDS, 10 mM NaF, 10 mM Na_2_HPO_4_, pH 7.4) plus HALT protease inhibitor cocktail (Thermo Fisher) and were cleared (15,000 rpm, 20 min), normalized to equal protein concentration, and denatured in 2 × SDS-sample buffer. Immunoblotting with appropriate primary antibodies (1:1000; TH (PA1–4679, Life Technologies); phospho-TH Ser40 (36–8600, Life Technologies); FFA4 (100364, GeneTex, Irvine, CA); and β-actin (3700S, Cell Signaling Technology) overnight at 4 °C was followed by visualized with HRP-conjugated secondary antibody on a ChemiDoc XLS+ (Bio-Rad, Hercules, CA). Each blot was repeated in 3–5 individual experiments as noted.

### 2.10. Cytotoxicity assay

Cell viability and cytotoxicity was measured by MTT assay (Biotium, Fremont, CA), as described previously [26]. Briefly, tissue or cells were treated as described and 10 μL of MTT solution was added for 4 h at 37 °C and followed by addition of DMSO for 10 min. Absorbance was measured at 570 nm using a Tecan Infinite M200.

### 2.11. cAMP formation

Cellular cAMP formation was measured by ELISA (Cayman Chemical, Ann Arbor, MI). Briefly, 1.0 × 10^5^ cells were grown in 12 well plates for 24 h, then starved 16 h and incubated with 100 μM 3-isobutyl-1-methylxanthine (IBMX) for 5 min prior to addition of drugs as indicated in the figure legend. cAMP was quantified at 405 and 420 nm on a Tecan Infinite M200 and expressed as a percentage of the maximum forskolin response.

### 2.12. Data analysis

Immunoblots were quantified by densitometric analysis using NIH Image J (Bethesda, MD) with integrated density standardized to the loading control, and graphed using GraphPad Prism 10 (San Diego, CA). Data are expressed as mean ± SD for representative experiments repeated at least three independent times. All quantitative experiments are also performed in triplicate biological replicates. Where not visible, error bars fall within the symbol size. Statistical analysis was performed in Graph Pad 10 using a one-way analysis of variance and post-hoc Bonferonni’s analysis, unless otherwise noted in the figure legends. Statistical significance is represented as a single symbol for p < 0.05, a double symbol for p < 0.01, and a triple symbol for p < 0.001, as noted in the figure legends.

## 3. Results

### 3.1. FFA4 is expressed in rat dorsal striatum and in DA-synthesizing PC12 pheochromocytoma cells

**Fig. 1.**
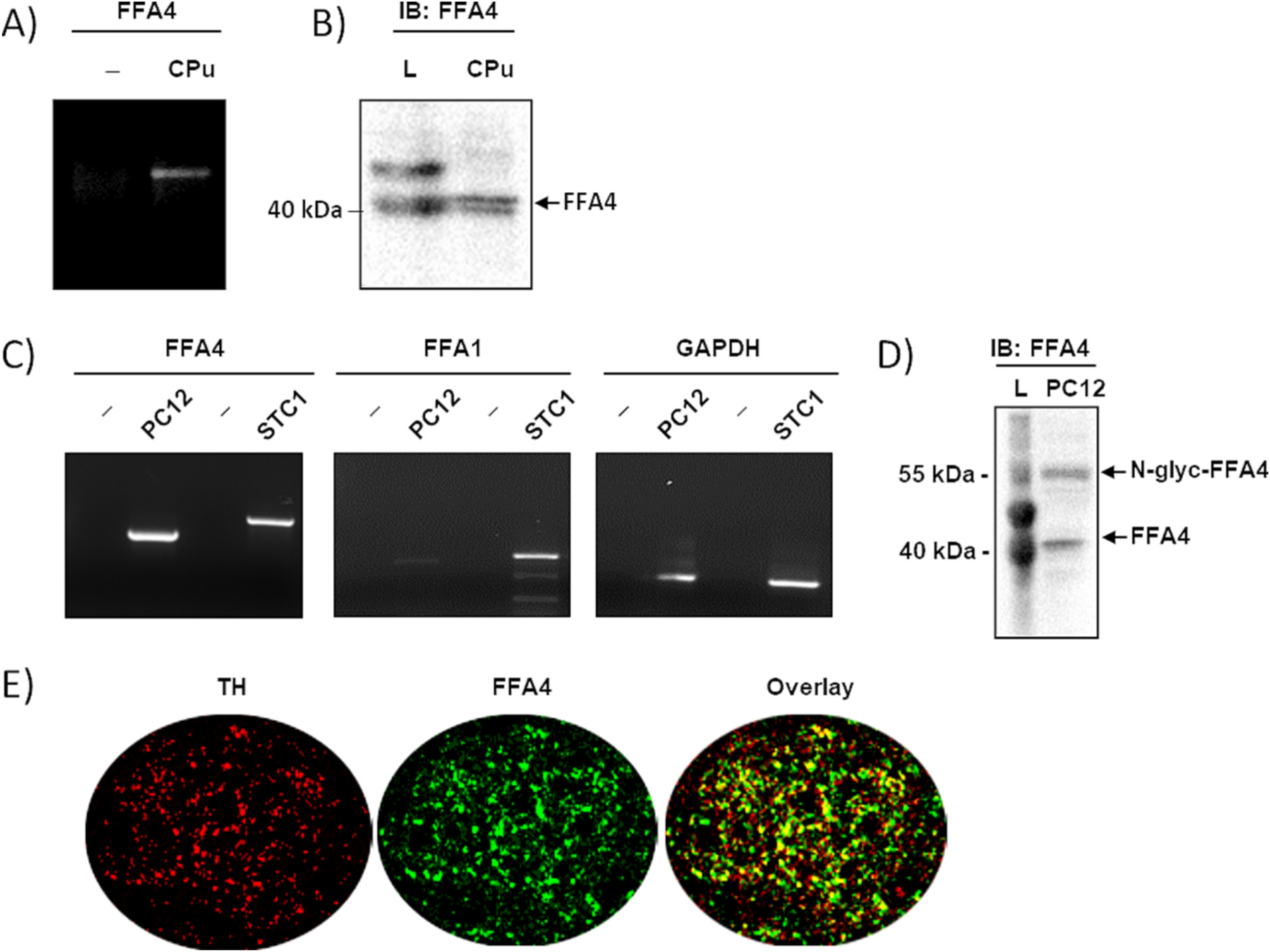
Expression of FFA4 in PC12 and rat striatum. (A-B) FFA4 mRNA and protein are expressed in rat CPu as determined by RT-PCR (A) and IB (B), respectively. – and L denote water control and protein ladder, respectively. (C) FFA4, but not FFA1 mRNA is expressed in rat PC12 cells as determined by RT-PCR. Murine STC1 cells, which are known to express both FFA receptors are used as a positive control. GAPDH is also a positive control for the PCR templates. (D) Native and glycosylated FFA4 protein are expressed in PC12 lysates as determined by IB. (E) Tyrosine hydroxylase (TH) and FFA4 expression in rat striatum as determined by immunofluorescence reveals substantial colocalization (overlay). Each experiment was repeated at least 3 times.

FFA4 has recently been described to be prevalently expressed in over 60 % of dopaminergic neurons, where it is functionally active and regulates midbrain behaviors including food and water intake, and modulation of the dopaminergic reward system [25]. Using RT-PCR and immunoblotting, here, we reveal the expression of FFA4 transcript and protein in rat caudate-putamen (i.e., dorsal striatum) tissue (Fig. 1A and B), which receives dopaminergic input from the substantia nigra pars compacta to regulate, in part, motor control and coordination. FFA4 was also expressed in the dopamine-synthesizing rat pheochromocytoma cell line PC12 (Fig. 1C and D), which is commonly used to study dopamine biosynthesis and signaling. We did not detect robust expression of FFA1 transcript in PC12 cells (Fig. 1), although it has previously been reported to be expressed in the mouse striatum [32,33]. Finally, immunofluorescent imaging demonstrated that FFA4 is co-localized with TH in the rat caudate-putamen (Fig. 1E).

### 3.2. FFA4 agonism increases phosphorylation of TH at serine-40 in PC12 cells and rat striatal minces

**Fig. 2.**
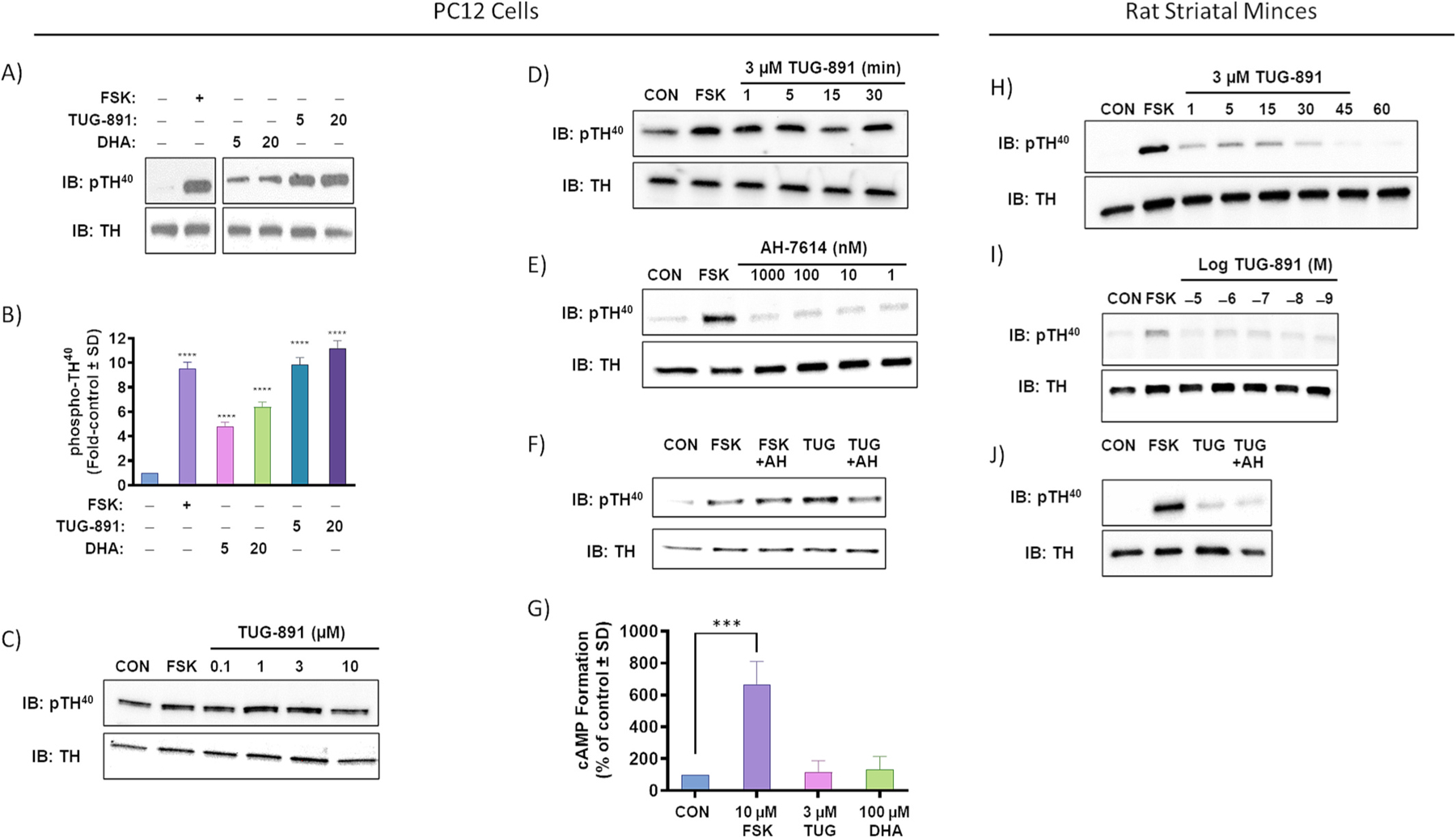
Effects of FFA4 agonists DHA and TUG-891 on phosphorylation of TH-Ser40 in PC12 cells and *ex vivo* rat striatal minces. (A, top panel) Treatment of PC12 cells with the endogenous ω3 FFA agonist DHA (100 μM) or the synthetic selective FFA4 agonist TUG-891 (3 μM) robustly increase phosphorylation of TH-Ser^40^ at 5 and 20 min following their addition, as demonstrated by IB. Forskolin (FSK, 10 μM, 20 min) is included as a positive control. (A, bottom panel) Blots were stripped of IgG and reprobed for total TH to ensure equal protein loading, as demonstrated in all subsequent blots (N = 3). (B) Chemiluminescent signal was quantified as pTH/TH using a Bio-Rad XRS camera and ImageLab software. Bars represent pTH/TH for each condition and are expressed as fold-increase over vehicle-control (lane 1). **** denotes p < 0.0001 versus vehicle-control (lane 1). (C) After 5 min, TUG-891 increases phosphorylation of TH-Ser^40^ in a concentration-dependent manner, an effect that peaks at 3 μM (N = 3). (D) Time dependency of TUG-891 (3 μM) (N = 3–5) (E) The selective non-competitive FFA4 antagonist AH-7614 (1 nM–1 μM) did not significantly alter phosphorylation of TH-Ser^40^. AH-7614 (1 μM) decreases but does not fully inhibit TUG-891 (3 μM)-induced phosphorylation of TH-Ser^40^ but has no effect on FSK (10 μM)-induced phosphorylation of TH-Ser^40^ (N = 3). (G) Neither TUG-891 (3 μM) nor DHA (100 μM) increased cAMP formation after 30 min, unlike that seen with FSK (10 μM). *** denotes p = 0.0003. (H) Treatment of rat striatal minces with TUG-891 (3 μM) increases phosphorylation of TH-Ser^40^, an effect that peaks at 5–15 min following its addition. 1 μM FSK is used as a positive control (N = 3). (I) After 5 min, TUG-891 increases phosphorylation of TH-Ser^40^ in rat striatal minces in a concentration-dependent manner, an effect that peaks at 1 μM. (J) AH-7614 (1 μM) decreases but does not fully inhibit TUG-891 (3 μM)-induced phosphorylation of TH-Ser^40^ in rat striatal minces (N = 3).

GPCR agonism increases intracellular second messengers that lead to activation of PKA, PKC, and ERK1/2, which in turn regulate phosphorylation and downstream catalytic activation of TH. PKA-mediated phosphorylation of Ser^40^ in the N-terminal regulatory region of TH is the primary driver of catalytic activity, and this residue can regulate phosphorylation of the other sites, including Ser^31^ [11]. To begin to dissect a role for FFA4 on TH activation, we assessed phosphorylation of TH-Ser^40^ in dopamine synthesizing PC12 cells, which are commonly used to characterize effects on dopamine function, in the presence of the endogenous ω3 FFA4 agonist docosahexaenoic acid (DHA) as well as the selective synthetic FFA4 agonist TUG-891 [28]. As expected, the adenylyl cyclase activator forskolin (FSK, 10 μM, 20 min), used here as a positive control, facilitated significant phosphorylation of TH-Ser^40^, while a physiologically relevant concentration of DHA (100 μM), which represents the low-end concentration of circulating DHA [34], as well as TUG-891 (3 μM) also significantly increased TH-Ser^40^ phosphorylation at both 5 and 20 min following their addition (Fig. 2A and B). We did not detect significant effects of DHA or TUG-891 at TH-Ser^31^ phosphorylation (data not shown). We further probed the concentration (Fig. 2C) and time (Fig. 2D) dependence of the stimulatory effects of TUG-891 on TH-Ser^40^, and results show a peak stimulation of TH-Ser^40^ at 3 μM and 5 min. Next, we examined the impact of the selective non-competitive FFA4 antagonist AH-7614 [35] on TH-Ser^40^ phosphorylation. While AH-7614 alone had no significant effect on TH-Ser^40^ phosphorylation at concentrations up to 1 μM (Fig. 2E), 1 μM AH-7614 significantly decreased, but did not fully inhibit TH-Ser^40^ phosphorylation induced by TUG-891 (Fig. 2F), suggesting non-FFA4 dependent effects may also occur with the agonist. The 1 μM concentration used here was based on its IC_50_ of ~100 nM [35], however, the lack of full blockade here may also be due to the non-competitive nature of the antagonist, which may be surmountable by 3 μM TUG-891. Since phosphorylation of TH-Ser^40^ is primarily driven by cAMP and other cyclic nucleotides that predominantly, but not exclusively activate PKA [7,11], we assessed cAMP formation in PC12 cells in the presence of TUG-891 (3 μM) and DHA (100 μM). Surprisingly, we found neither FFA4 agonist able to increase cAMP formation (Fig. 4G) in these cells.

Given these effects in PC12 cells, we next assessed the effects of TUG-891 in *ex vivo* minces of rat striatal tissue where a time course using 3 μM concentration of TUG-891 exhibited peak TH-Ser^40^ phosphorylation efficacy at 15–30 min following agonist addition (Fig. 2H). In *ex vivo* minces, the agonist exhibited strongest TH-Ser^40^ phosphorylation in the 100 nM-1 μM range (Fig. 2I). Finally, the effect of TUG-891 in rat striatal minces was decreased, but as in PC12 cells, not fully inhibited by the non-competitive FFA4 agonist AH-7614 (Fig. 2J).

### 3.3. FFA4 agonism provides neuroprotection against 6-OHDA-induced toxicity

**Fig. 3.**
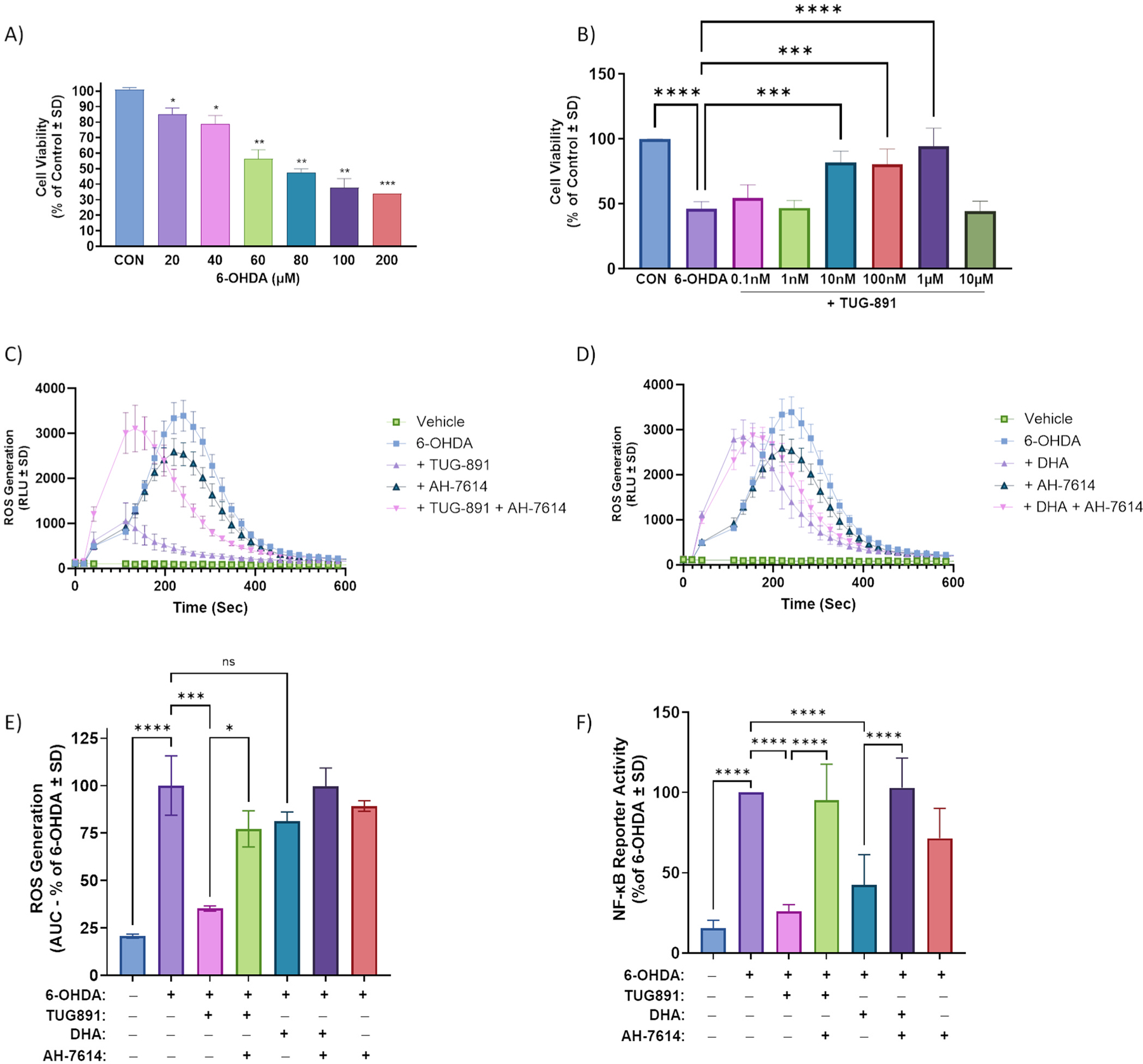
FFA4 agonism reduces 6-OHDA-induced cytotoxicity, oxidative stress, and NF-κB transcriptional activity in PC12 cells. (A) 6-OHDA induces PC12 cell death in a concentration-dependent manner as demonstrated by MTT cell viability. * denotes p < 0.05, ** denotes p < 0.01, and *** denotes p < 0.001 compared to vehicle-control (CON). N = 3 performed in triplicate. (B) Incubation of PC12 cells with the FFA4 agonist TUG-891 in the presence of 100 μM 6-OHDA attenuates cytotoxicity in a concentration-dependent, bell-shaped manner, with significant protection observed at 10 nM, 100 nM, and 1 μM concentrations. *** denotes p < 0.001 and **** denotes p < 0.0001, relative to the indicated comparison. N = 4 performed in triplicate. (C,E) 6-OHDA (100 μM) induces significant ROS generation in PC12 cells as measured by the ROS-sensitive luminescent probe L-012 (blue squares). The effect of 6-OHDA was significantly reduced by 30 min pretreatment with TUG-891 (3 μM) (purple triangle), while the suppressive effects of TUG-891 were reversed by the presence of AH-7614 (10 μM) (pink triangle). AH-7614 alone had no significant effect on 6-OHDA-induced ROS generation (blue triangle). Data in (E) represent the average AUC of each curve in (C), from an N = 3, each performed in triplicate. * denotes p < 0.05, *** denotes p < 0.001 and **** denotes p < 0.0001, relative to the indicated comparison. (D,E) The effect of 6-OHDA was not significantly affected by 30 min pretreatment with DHA (100 μM) (purple triangle) or AH-7614 (blue triangle). Data in (E) represent the average AUC of each curve in (D), from an N = 3, each performed in triplicate. (F) 6-OHDA (1 μM) induces significant NF-κB transcriptional activity in PC12 cells, as measured by the Ready-To-Glow NF-κB luciferase reporter assay. Both TUG-891 (3 μM) and DHA (100 μM) significantly reduced 6-OHDA-induced NF-κB transcriptional activity and the effects of both were reversed upon treatment with AH-7614 (50 μM, 30 min prior to agonist), which had no significant effect on its own. *** denotes p < 0.001 and **** denotes p < 0.0001, relative to the indicated comparison. N = 5–6 performed in triplicate.

6-Hydroxydopamine (6-OHDA) is a selective catecholaminergic neurotoxin structurally analogous to DA, and which is actively transported into dopaminergic and noradrenergic nerve terminals via their respective transporters. Once internalized, 6-OHDA undergoes rapid auto-oxidation, facilitating the generation of ROS, including H_2_O_2_, as well as the highly oxidative DA semiquinone and quinone intermediates [36,37], which facilitate oxidative cytotoxicity. To determine if FFA4 agonism protects against 6-OHDA-induced cytotoxicity, we first assessed the dose-dependency of 6-OHDA mediated cytotoxicity via MTT in PC12 cells and observed a plateau in the effect at 100–200 μM (Fig. 3A). Next, we evaluated the effects of increasing concentrations of TUG-891 (0.1 nM-10 μM) on protection against cytotoxicity induced by 100 μM 6-OHDA. TUG-891 decreased cytotoxicity induced by 6-OHDA in a concentration-dependent, bell-shaped fashion with no effect at 0.1–1 nM and statistically significant, near complete protection at 10 nM-1 μM, which was lost at the highest concentration used (10 μM) (Fig. 3B).

### 3.4. FFA4 agonism decreases ROS generation and NF-κB activity in PC12 cells

ROS formed by dysregulated mitochondria and dopamine metabolism are central drivers of dopamine cell death as well as neurodegeneration in PD, contributing to oxidative damage of lipids, proteins and DNA in vulnerable DA neurons. In particular, dopaminergic neurons in the substantia nigra exhibit heightened susceptibility to oxidative stress due to their high metabolic rate, iron content and dopamine autoxidation, amplifying ROS-mediated degeneration in PD [38–41]. Moreover, redox-sensitive NF-κB activity promotes transcription of proinflammatory cytokines and proapoptotic mediators, directly linking the ROS-NF-κB axis to dopaminergic cytotoxicity [42,43]. Previously, we have shown that FFA4 agonism or its overexpression in macrophages markedly suppresses PMA-induced ROS generation and LPS-driven NF-κB activity [26]. To begin to investigate whether FFA4 similarly modulates ROS production in dopamine synthesizing cells, we evaluated the effects of TUG-891 and DHA on 6-OHDA-induced ROS generation in PC12 cells. Addition of 6-OHDA (100 μM) caused a rapid and robust increase in ROS generation (Fig. 3C and E). This effect was significantly decreased in the presence of the FFA4 agonist TUG-891 (3 μM), but not the FFA4 antagonist AH-7614 (10 μM) (Fig. 3C and E). Moreover, the ROS suppressive effects of TUG-891 were lost in the presence of AH-7614, demonstrating that the ROS modulation is FFA4 dependent (Fig. 3C and E). Interestingly, the endogenous FFA4 agonist DHA (100 μM) only had a small but insignificant left-shifting effect on 6-OHDA-induced ROS generation, similar to that seen in its presence with AH-7614 and did not reduce ROS as did TUG-891, suggesting that in this assay, it does not engage FFA4 signaling cascades that modulate ROS formation (Fig. 3D and E). Next, we used an NF-κB activity reporter assay to assess the effects of TUG-891 and DHA on 6-OHDA-induced NF-κB–dependent transcriptional activity. Here, both TUG-891 (3 μM) and DHA (100 μM) significantly inhibited 6-OHDA-induced NF-κB activation, and the effect of both agonists was reversed in the presence of AH-7614 (50 μM), demonstrating an FFA4-mediated effect (Fig. 3F).

### 3.5. FFA4 agonism improves motor control and provides neuroprotection in rat 6-OHDA model of PD

**Fig. 4.**
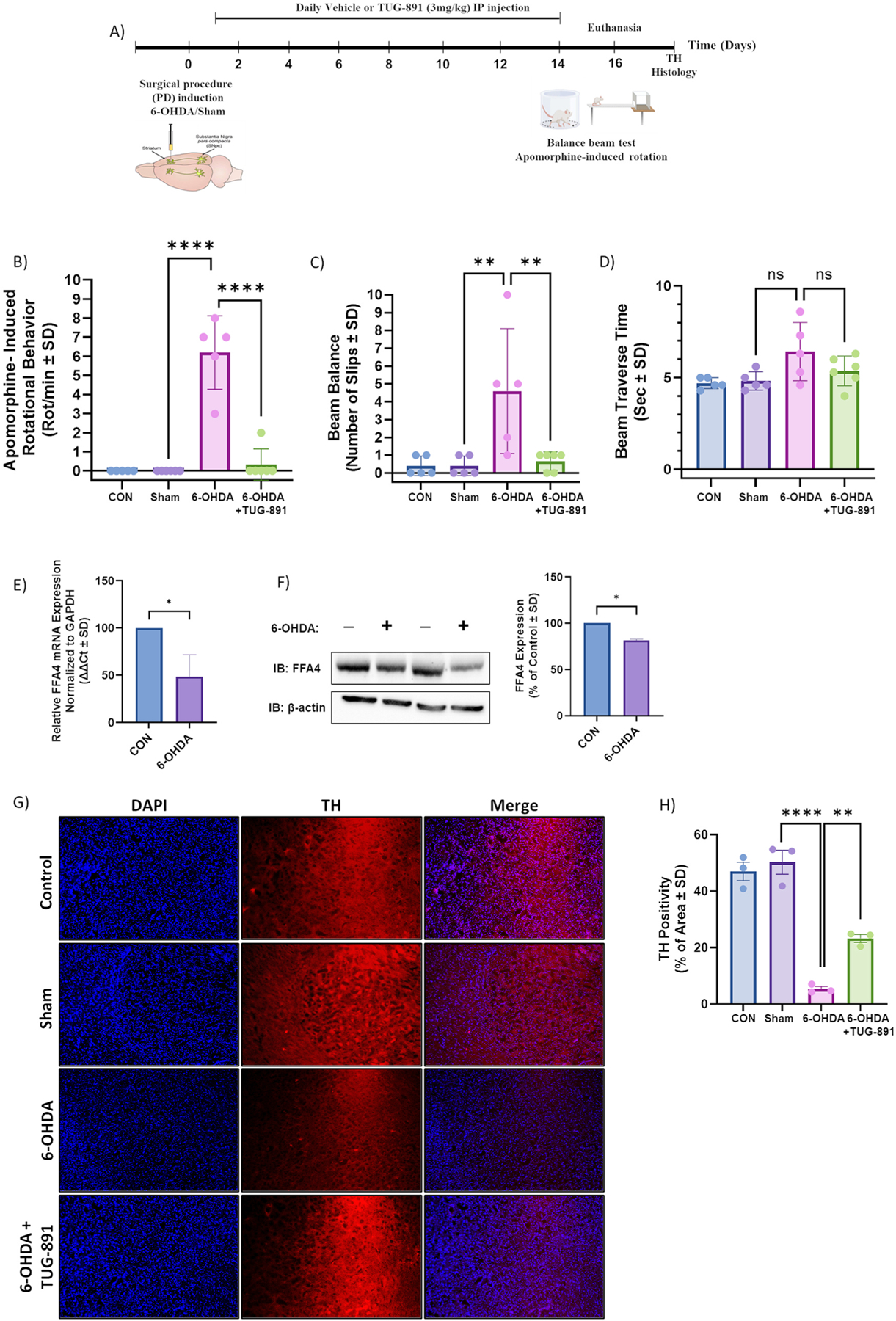
FFA4 agonism by TUG-891 mitigates 6-OHDA-induced motor dysfunction and dopaminergic neuronal loss in the 6-OHDA rat model of PD. (A) Schematic of unilateral 6-OHDA injection and treatment paradigm. Animals were randomized to the four cohorts. Control (CON) animals (N = 5) received vehicle IP injections in place of TUG-891 (N = 8) and Sham animals (N = 6) received surgical lesioning with 0.9 % saline in place of 6-OHDA and also received vehicle IP injections. (B) Unilateral 6-OHDA lesioning of rats (6 μg/μl in 0.9 % saline containing 0.2 % ascorbic acid, N = 8) induced significant contralateral rotational behavior upon injection of the dopamine agonist apomorphine (3 mg/kg, IP). This effect was significantly inhibited in lesioned rats treated daily for 14 days with TUG-891 (3 mg/kg, IP) beginning one day following lesioning. (C) Rats were assessed for the number of slips from the beam in a beam balance test and 6-OHDA lesioned rats exhibited significantly higher number of slips, an effect that was significantly inhibited in animals treated with TUG-891. (D) There was no observable difference in the time to traverse the entire length of the beam. (E) 6-OHDA (100 μM) reduces FFA4 transcript expression in rat striatal minces as measured by real-time qPCR. * denotes p < 0.05 versus vehicle-control (CON) via Student’s *t*-test. (D) 6-OHDA (100 μM) reduces FFA4 protein expression in 6-OHDA-lesioned rats as measured by IB. * denotes p < 0.05 versus vehicle-control (CON) via Student’s *t*-test. (G-H) Immunofluorescence (G) and quantification (H) of fixed cryopreserved striatal tissue for TH and DAPI demonstrates significant loss of TH and nuclei staining in 6-OHDA-lesioned animals, an effect that is significantly restored in lesioned animals treated with TUG-891. ** denotes p = 0.01, **** denotes p < 0.0001, relative to the indicated comparison.

Given our results from PC12 cells and striatal minces that suggest a neuroprotective role of FFA4, we assessed the effects of TUG-891 in a unilateral 6-OHDA lesion rat model of PD. As shown in Fig. 4A, rats were unilaterally lesioned with 6-OHDA on day 0 and treated with daily IP injections of TUG-891 (3 mg/kg) or vehicle for 14 days, following which, motor coordination and balance was assessed by apomorphine-induced rotation as well as beam balance test. TH immunofluorescence was evaluated after euthanasia following motor assessments. Unilateral 6-OHDA injection in this model elicited significant apomorphine (3 mg/kg, IP)-induced rotational behavior, which was lacking in vehicle-treated control and in animals that underwent sham surgery with saline injection in place of 6-OHDA (Fig. 4B). Lesioned animals that were treated with TUG-891 for 14 days exhibited a near-complete absence of apomorphine-induced rotational behavior (Fig. 4B). These results were mirrored by beam balance tests, in which the number of slips/falls as the animal traversed the beam was significantly reduced in 6-OHDA-lesioned animals treated with TUG-891 compared to lesioned animals that were untreated (Fig. 4C). Although TUG-891 treatment tended to decrease the total time it took animals to traverse the balance beam (Fig. 4D), the difference was not significant. Quantitative real-time PCR in *ex vivo* rat striatal minces treated with 6-OHDA reveals significant reduction of FFA4 expression (Fig. 4E), consistent with the colocalization of FFA4 with TH-positivity (Fig. 1E). This effect was also demonstrated in the rat 6-OHDA-lesion model, where 6-OHDA lesioning significantly reduced the expression of FFA4 protein (Fig. 4F). Finally, to determine the effects of TUG-891 on neuroprotection from 6-OHDA-induced striatal neuron death, we assessed TH expression via immunofluorescence. While 6-OHDA lesioning significantly decreased both cell nuclei (i.e., DAPI) and TH expression, lesioned animals treated with TUG-891 demonstrated preserved TH positivity and total cell numbers (Fig. 4G and H).

## 4. Discussion

Here for the first time, we provide evidence that FFA4 is expressed in TH-expressing dopaminergic neurons of the rat striatum and in dopamine-synthesizing PC12 cells, and that FFA4 activation confers neuroprotective effects against 6-OHDA-induced toxicity, and alleviates motor symptoms in a rat model of PD. Our RT-PCR, immunoblotting, and immunofluorescence analyses confirm the presence of FFA4 in TH-positive neurons, and suggest that FFA4 modulates dopaminergic function, perhaps via regulation of ROS and anti-inflammatory effects via inhibition of NF-κB.

Functionally, FFA4 agonism enhanced phosphorylation of TH at Ser^40^ in both PC12 cells and striatal tissue, indicating activation of the rate-limiting step in dopamine synthesis. Importantly, this effect was observed with the synthetic agonist TUG-891 at low micromolar concentrations and was decreased by the selective FFA4 antagonist AH-7614, confirming receptor-specific signaling. These findings suggest that FFA4 activation can directly influence dopamine synthesizing enzymatic activity, potentially supporting dopamine synthesis under both basal and conditions mimicking oxidative stress (e.g., 6-OHDA). Interestingly, we observe that the effects of TUG-891 on phosphorylation of TH-Ser^40^ in both PC12 cells and rat striatal minces is decreased but not fully inhibited by the FFA4 antagonist AH-7614 (Fig. 2F and I). While AH-7614 is a highly selective FFA4 antagonist, it acts as a non-competitive antagonist, which may limit its ability to completely block receptor-mediated signaling in the presence of TUG-891, particularly under conditions of higher agonist concentration or prolonged exposure. In addition, while TUG-891 exhibits excellent selectivity for hFFA4 over hFFA1 with respect to both Gα_q/11_-Ca^+2^ signaling (pEC_50_ of 7.02 ± 0.09 versus 5.3 ± 0.04; 52-fold selectivity for FFA4) and β-arrestin-2 recruitment (pEC_50_ of 7.22 ± 0.06 versus 4.76 ± 0.29; 288-fold selectivity for FFA4), this selectivity was significantly decreased at the mouse ortholog of the receptor, resulting in 3-fold selectivity for FFA4 at Ca^+2^ signaling (pEC_50_ of 6.89 ± 0.04 versus 6.41 ± 0.08) and 61-fold selectivity for FFA4 at β-arrestin-2 recruitment (pEC_50_ of 7.77 ± 0.09 versus 5.92 ± 0.16) [28]. While it is unknown which signaling pathways regulate FFA4-mediated effects seen herein, it is not outside the realm of possibility that TUG-891 also demonstrates lower selectivity at the rat receptor and some of these effects may be due to FFA1 engagement. While we did not detect FFA1 on PC12 cells, it is expressed in mouse striata [33]. Moreover, FFA1 is expressed in other neuronal circuits and may influence dopaminergic function indirectly, for example via modulation of glutamatergic or other neuromodulatory inputs, potentially contributing to the observed effects. Finally, additional compensatory signaling pathways downstream of GPCR activation, including crosstalk between PKA, PKC, and ERK1/2, all of which converge on the N-terminal phosphorylation sites of TH [9,44,45], may also maintain partial TH phosphorylation even in the presence of AH-7614, suggesting that multiple mechanisms may interact to regulate TH activation. Together, these considerations indicate that while FFA4 is a major mediator of TUG-891-induced TH phosphorylation, other receptor interactions and signaling complexities may contribute to the partial persistence of this effect.

FFA4 agonism conferred robust neuroprotection against 6-OHDA-induced cytotoxicity and oxidative stress in the form of ROS generation. In PC12 cells, TUG-891 preserved TH phosphorylation, reduced ROS generation and suppressed NF-κB activity, highlighting a likely mechanism through which FFA4 signaling mitigates oxidative and inflammatory pathways implicated in PD pathophysiology. While the endogenous agonist DHA produced robust TH phosphorylation and markedly suppressed NF-κB transcriptional activity in PC12 cells similar to TUG-891, it failed to significantly attenuate 6-OHDA-induced ROS generation, in contrast to the synthetic agonist (Fig. 3). Interestingly, we have previously shown that both TUG-891 and DHA robustly inhibit PMA-induced ROS generation in macrophages [26], indicating that the nature of the ROS-inducing stimulus and the cell type-specific ROS generating machinery may influence FFA4-dependent antioxidant responses. This apparent discrepancy may also reflect several mechanistic and experimental factors. First, the ROS assay measures acute oxidative responses that peak within minutes of 6-OHDA exposure, whereas the NF-κB reporter assay involves a 6-hour agonist incubation, while TH phosphorylation was assessed at 5 and 20 min after agonist addition. Hence, the temporal dynamics of signaling may underlie the observed differences. Second, DHA is a long-chain polyunsaturated FFA that readily incorporates into plasma and intracellular membranes, where it may exert non-receptor-mediated biophysical or antioxidant effects independent of FFA4 activation. In contrast, TUG-891 is a synthetic, nonlipid small molecule with higher receptor specificity and minimal expected membrane partitioning, allowing for more direct FFA4-mediated signaling. Additionally, DHA’s intracellular accumulation may limit its effective engagement of plasma membrane FFA4 receptors during the brief time window of ROS generation, whereas TUG-891 likely maintains consistent receptor occupancy and signaling efficacy under these conditions. On the contrary, in adipocyte models, FFA4 has recently been shown to be expressed more predominantly on intracellular organelle membranes compared to the cell surface [46], and if this is the case in other cell types including neurons and PC12 cells, the effects of DHA would be expected to be more pronounced on intracellular FFA4 compared to TUG-891, and may not be as readily inhibited by antagonists like AH-7614. Finally, previous work has demonstrated that distinct FFA4 agonists, including TUG-891 and ω3 FFA like DHA, can act as biased agonists at FFA4-linked signal cascades, preferentially engaging distinct Gα subunits, as well as β-arrestins [19,47,48]. Hence, it may be plausible that DHA and TUG-891 differentially bias FFA4 signaling toward distinct intracellular cascades, with TUG-891 more effectively coupling to pathways that suppress acute ROS formation, whereas DHA disfavors this and leans towards cascades that drive the TH and transcriptional mechanisms. Further work in our laboratory will tease out these intracellular cascades. Together, these factors suggest that while both agonists can activate downstream signaling through FFA4, their kinetic profiles, physicochemical properties, and subcellular distribution may differentially influence their capacity to modulate acute oxidative stress.

Our finding that neither TUG-891 or DHA was able to increase cAMP was surprising, given the phosphorylation of TH-Ser^40^ is most strongly influenced by cyclic nucleotides that signal predominantly via PKA, but may also include other kinases such as ERK1/2 [7,9,11,45]. While ω3 FFA, including DHA and EPA, have been shown to facilitate FFA4-Gα_S_ signaling [19,47] in addition to the predominant Gαq/11 signaling, this was not the case in PC12 cells, as evidenced by the lack of ability of either TUG-891 or DHA to induce cAMP formation. This suggests that in PC12 cells, FFA4 may preferentially couple to non-Gαs pathways, potentially reflecting ligand- or cell type-dependent biased signaling, as previously described [19,47,48]. Given that FFA4 activation is strongly linked to ERK1/2 signaling, and that ERK1/2 can directly phosphorylate TH at Ser^40^, it is plausible that the observed TH phosphorylation following TUG-891 and DHA stimulation occurs through an ERK1/2-dependent mechanism rather than via cAMP/PKA. Our prior work, and that of others, has shown that FFA4 engages ERK1/2 both through classical Gαq/11–PKC signaling and β-arrestin–dependent pathways [26,30,31,49,50], raising the possibility that TUG-891, and to a lesser extent DHA, may bias FFA4 signaling toward ERK1/2 activation, leading to TH phosphorylation independent of canonical cAMP pathways. Further exploration of these mechanisms may elucidate how endogenous versus synthetic FFA4 agonists differentially engage receptor signaling networks and ultimately influence dopaminergic neuron function.

Our *in vivo* results suggest that FFA4 activity confers significant histological and functional neuroprotection in the rat 6-OHDA model of PD. TUG-891 reduced apomorphine-induced rotations and improved performance on beam balance, consistent with preservation of nigrostriatal motor function, despite a decreased expression of FFA4. Motor improvement was paralleled by preservation of TH immunoreactivity, suggesting that FFA4 agonism mitigates dopaminergic cell loss and may support cell survival. Although the mechanisms by which FFA4 exerts these effects *in vivo* remain undefined, and our data do implicate a role for FFA4 in neuronal (e.g., TH expressing) cells, emerging evidence also highlight a potential role for glial FFA4 in modulating neuroinflammatory responses that may regulate to neurodegeneration. FFA4 is densely expressed in human and murine microglia [24], and in brain regions such as the hypothalamus, the receptor is primarily glial rather than neuronal [21]. In the nucleus accumbens, a distinct dopaminergic pathway than that studied here, FFA4 was densely localized to microglia, and agonism of FFA4 significantly decreased proinflammatory Iba-1, TNF-α, and IL-1β expression and strongly decreased both sickness- and anxiety-like behaviors triggered by systemic LPS and central TNF-α and IL-1β administration [24], establishing a significant role for glial FFA4 in mitigating neuroinflammation in dopaminergic systems. Similarly in the hippocampus, 6-week treatment with the ω3 FFA ALA significantly upregulated glial expression of FFA4, an effect that translated to reduced glial polarization and microglia-mediated inflammation, which were inhibited by AH-7614 [51]. Consistent with these results, FFA4 knockout (KO) mice exhibit significant microglial activation, prostaglandin synthesis and downstream neuroinflammation and hippocampal degeneration [52], suggesting a significant role for glial FFA4 in regulation of neuroinflammation and neuroprotection. Mechanistically, FFA4 has been shown to promote significant anti-inflammatory effects in both macrophages and glia via signaling through β-arrestin-2 partner proteins [23,26,53] and given the established anti-inflammatory actions of glial FFA4 in other dopaminergic and hippocampal circuits, it is plausible that similar mechanisms operate within the nigrostriatal pathway to mitigate toxin-induced degeneration. In addition, although direct studies of FFA4 in astrocytes are less abundant, there is also evidence that FFA4 signaling influences astrocyte function and extracellular proteolytic activity [22], pointing to the possibility that FFA4 may also regulate astrocyte responses in the injured CNS. However, given our findings that FFA4 activation produces rapid, minute-scale changes in TH phosphorylation and is colocalized with TH, the neuroprotective effects observed *in vivo* are unlikely to arise exclusively from astrocyte or microglial signaling. Rather, we presume a model in which both neuron-intrinsic FFA4 and glial modulatory mechanisms contribute to the overall protective response. Further work is underway in our laboratory to define the cell-specific contributions as well as cell signaling cascades involved.

Sex differences in PD are well documented and gonadal steroids are well-described to influence neurodegenerative diseases, including PD [54–56]. Along with age, male sex is a significant risk factor for development of PD in humans [56], while estrogens and selective estrogen receptor modulators (SERM) have been shown to be protective of dopaminergic neurons in rodent models of PD [57–59]. Given this protective effect of estrogens and the consequential variability that arises in rodent models of PD in female animals, we have only examined the role of FFA4 in male animals in our initial studies described here. Nevertheless, sex differences in PD susceptibility, progression, and response to interventions are well documented and future studies will be important to examine FFA4-mediated effects in female animals to determine whether similar dopaminergic regulation and neuroprotective mechanisms occur, thereby providing a more comprehensive understanding of sex-dependent influences in PD. This is particularly significant given the expression of FFA4 on pituitary gonadotropes and gonadotrope-derived cell lines, as well as the functional role of FFA4 in regulating FSH and LH secretion [60–62]. Therefore, evaluating FFA4 effects in females will be essential to determine whether gonadal hormone interactions with FFA4 modify dopaminergic vulnerability or FFA4-agonist responsiveness. Such studies will ultimately clarify whether FFA4-targeted interventions have sex-specific efficacy profiles, an increasingly important consideration for translational relevance in PD.

Collectively, our findings support a model in which FFA4 activation exerts multifaceted neuroprotective effects within DA synthesizing cells through simultaneous regulation of oxidative, inflammatory, and TH phosphorylation-linked pathways. By promoting TH phosphorylation and attenuating ROS and NF-κB activity, FFA4 signaling may sustain DA synthesis and neuronal viability under neurotoxic stress conditions. Given the established link between chronic neuroinflammation, oxidative stress, and dopaminergic degeneration in PD, targeting FFA4 represents a promising therapeutic avenue for modulating these convergent pathogenic processes. Integrating FFA4-targeted strategies with FFA1 agonism, previously demonstrated to promote dopaminergic function, decrease inflammation and provide neuroprotection in dopaminergic neurons and mouse models of PD [33], could produce complementary or synergistic neurorestorative effects. Future studies should also explore the *in vivo* relevance of these findings using genetic deletion models, as well as delineate the contribution of ERK1/2 and other kinase pathways, such as CaMKII or Akt, to TH regulation and neuroprotection. Finally, given the pleiotropic roles of FFA4 across metabolic and immune (e.g., glia) systems, understanding its signaling dynamics in the nigrostriatal circuit could illuminate novel links between lipid metabolism, inflammation, and dopamine neuron survival, offering new therapeutic insights for neurodegenerative diseases such as PD. Here, for the first time, we have established FFA4 as a novel modulator of dopamine-synthesizing cell survival and function, positioning this GPCR and its endogenous lipid ligands as a potentially promising therapeutic target in PD.

## Data Availability

All data generated or analyzed in this study are included in this published article.

## Abbreviations:

ALA: α-linolenic acid
DA: dopamine
DHA: docosahexaenoic acid
EPA: eicosapentaenoic acid
FFA: free-fatty acid
FFA4: free-fatty acid receptor-4
FFAR: free-fatty acid receptor
FSK: forskolin
H_2_O_2,_: hydrogen peroxide
IBMX: 3-isobutyl-1-methylxanthine
MFB: median forebrain bundle
MTT: 3-(4,5-dime-thylthiazol-2-yl)-2,5-diphenyltetrazolium bromide
6-OHDA: 6-hydroxydopamine
PCR: polymerase chain reaction
PD: Parkinson’s disease
ROS: reactive oxygen species
SNc: substantia nigra pars compacta
TH: tyrosine hydroxylase
